# Postpartum Depression: Interacting Biological Pathways and the Promising Validation of Blood-Based Biomarkers

**DOI:** 10.3390/jcm14124286

**Published:** 2025-06-16

**Authors:** Livia Ciolac, Elena Silvia Bernad, Anca Tudor, Dumitru-Răzvan Nițu, Florina Buleu, Daian-Ionel Popa, Teodora Toc, Carmen Haivas, Marius Lucian Craina

**Affiliations:** 1 Doctoral School, Faculty of General Medicine, “Victor Babes” University of Medicine and Pharmacy, 300041 Timisoara, Romania; livia.ciolac@umft.ro (L.C.); daian-ionel.popa@umft.ro (D.-I.P.); teodora.toc@umft.ro (T.T.); 2 Department of Obstetrics and Gynecology, Faculty of Medicine, “Victor Babes” University of Medicine and Pharmacy, 300041 Timisoara, Romania; bernad.elena@umft.ro (E.S.B.); nitu.dumitru@umft.ro (D.-R.N.); mariuscraina@umft.ro (M.L.C.); 3 Clinic of Obstetrics and Gynecology, “Pius Brinzeu” County Clinical Emergency Hospital, 300723 Timisoara, Romania; 4 Center for Laparoscopy, Laparoscopic Surgery, In Vitro Fertilization, “Victor Babes” University of Medicine and Pharmacy, 300041 Timisoara, Romania; 5 Department of Biostatistics and Medical Informatics, “Victor Babeș” University of Medicine and Pharmacy, 300041 Timisoara, Romania; 6 Department of Cardiology, “Victor Babes” University of Medicine and Pharmacy, 300041 Timisoara, Romania; florina.buleu@umft.ro; 7 Research Center for Medical Communication, “Victor Babes” University of Medicine and Pharmacy, 300041 Timisoara, Romania; 8 Department of Anatomy and Embryology, “Victor Babes” University of Medicine and Pharmacy, 300041 Timisoara, Romania; haivas.carmen@umft.ro

**Keywords:** postpartum depression, Edinburgh Postnatal Depression Scale (EPDS), screening, biomarkers, biological pathways, risk factors, preventive strategy, diagnosis

## Abstract

**Background/Objectives**: Postpartum depression (PPD), the most common and prevalent psychiatric disorder after birth, is a prevalent yet underdiagnosed psychiatric condition that remains insufficiently understood, particularly in terms of its biological basis. While epidemiological data are extensive, few studies have systematically investigated their underlying biological mechanisms. The purpose of this study was to explore the potential links between blood biomarker levels and postpartum depressive symptoms, contributing to the development of a unified biological model of PPD. **Methods**: We conducted a cross-sectional study between 2023 and 2025 at a tertiary academic hospital in Timisoara, Romania, involving 860 postpartum women recruited at hospital discharge (1–2 weeks after childbirth). The participants completed the Edinburgh Postnatal Depression Scale (EPDS) and provided peripheral blood samples, which were analyzed using standardized protocols. The blood levels of pregnancy-related hormones (estrogen and progesterone), vitamin D, biochemical markers of inflammatory response (white blood cell count, C-reactive protein, fibrinogen, neutrophil count, lymphocyte count, and ferritin), anemia indicators (hemoglobin, red blood cell count, hematocrit, and ferritin), thyroid hormones (TSH, FT3, and FT4) and markers of coagulation abnormalities (D-dimer, platelets, fibrinogen, APTT, and INR) were evaluated. The data were analyzed with JASP v0.19.3. The statistical methods included multivariate linear regression, the Kruskal–Wallis and Mann–Whitney U tests, and Spearman correlation, with significance set at *p* < 0.05. **Results**: The analysis revealed that postpartum depression (PPD) is associated with distinct biological profiles, reflecting the unique hormonal and physiological changes in the peripartum period. Significant associations were identified between EPDS scores and the levels of estrogen, progesterone, thyroid hormones (TSH, FT3, and FT4), inflammatory markers (CRP and ferritin), vitamin D, and coagulation parameters (APTT and INR). These findings support the notion that PPD has a multifactorial biological basis and highlight the potential of these biomarkers as early predictors of risk. **Conclusions**: Integrating biochemical assessments into postpartum care may enhance early identification and inform targeted preventive interventions, such as hormone monitoring, vitamin D and iron supplementation, or thyroid function correction.

## 1. Introduction

Peripartum depression represents a severe psychiatric disorder that remains insufficiently studied, clinically and experimentally, often goes undiagnosed, and typically begins during pregnancy or within the first four weeks after childbirth [1]. In the *Diagnostic and Statistical Manual of Mental Disorders, Fifth Edition* (DSM-5), peripartum depression is categorized as a Major Depressive Disorder, with peripartum onset, since approximately one-third of women with PPD experience symptom onset during pregnancy [2]. The majority of specialists believe that the beginning of clinical symptoms appears during pregnancy and up to one year after delivery [3]. Postpartum depression is the most prevalent psychiatric disorder during the peripartum period, affecting between 6.5% and 20% of postpartum individuals worldwide [4]. The World Health Organization, in 2008, identified major depressive disorder as the third most significant contributor driver to global incidence of illness and mortality, with projections suggesting it may become the leading cause by 2030 [5]. Moreover, studies estimate that around 50% of women with PPD remain undiagnosed [6,7]. Perinatal depression is linked to an increased likelihood of suicide among parents, the second most frequently occurring cause of postpartum mortality [8].

The prevalence of depression in women is double that found in men, with notable differences between genders in symptom presentation and likely contributing factors of major depressive disorder [9]. The variations in reproductive hormones between males and females have been suggested as a potential cause of these differences; however, additional contributing factors have also been identified [10]. These include the distinct patterns of stress response between men and women, comorbidity with anxiety disorders, and the greater genetic predisposition to major depressive disorder observed in women (42%) compared with men (29%) [10]. While men and women show similar likelihoods of experiencing recurrent depression [11,12], the prevalence of hormone-influenced episodes of depression, such as premenstrual dysphoric disorder, postpartum depression, and perimenopausal depression, suggests that fluctuating hormone levels have been linked to an elevated distinct risk in women [13]. Therefore, a growing genetic tendency toward depression could heighten women’s vulnerability to depressive episodes when faced with intense biological, psychological, and social stress, particularly during reproductive events [9].

A wide range of risk factors for postpartum depression has been investigated over the years. Historically, research has predominantly focused on psychosocial determinants, including a personal or family history of psychiatric disorders, particularly prior episodes of PPD [14], as well as low socioeconomic status, limited educational attainment, substance use, and insufficient social or partner support [15,16]. Obstetric variables, such as unplanned pregnancy, pregnancy complications, and mode of delivery, have also been examined as potential contributors [17]. While these factors remain clinically relevant, the primary focus of this research is on the biological risk factors which are involved in the etiology of PPD.

PPD is a common and debilitating condition that negatively impacts the well-being and development of both mothers and infants [6]. Postpartum depression has a detrimental effect on the mother, with suicide responsible for around 20% of postpartum mothers’ deaths [6,18], while also adversely affecting the developmental progress of infants across emotional, behavioral, and cognitive domains [19]. Additionally, a recent report found that a minimum of 5% of women experiencing peripartum depression experience depression that is resistant to treatment within one year of their PPD diagnosis, meaning they remain unresponsive to at least three different antidepressant treatments or a combination therapy involving one antidepressant and one antipsychotic within that year [20]. The most widely used methods for diagnosing peripartum depression are clinician-administered scales and self-report questionnaires [9]. Despite decades of research, no reliable or practical biological diagnostic tests for PPD have been developed, and there is no reliable screening method available to detect women at risk of postpartum depression [21]. A blood test is desirable to nonpsychiatric practitioners, who typically lack training in diagnosing or treating peripartum depression. Therefore, developing effective treatment strategies and a suitable medical approach for peripartum depression is an urgent priority. Regrettably, the complexity and variability of peripartum depression and the inability to pinpoint targeted biomarkers challenges the stratification of patients into more uniform clinical groups, making it challenging to determine the most effective therapeutic strategies for this condition. An objective and targeted laboratory-based tool could enhance the diagnostic accuracy of postpartum depression and facilitate individualized treatment. These findings, along with others, suggest that supporting postpartum depression diagnosis in primary care could considerably boost the effectiveness and timeliness of both diagnosis and therapeutic interventions. Specificity holds as much importance as reliability when it comes to making a proper diagnosis; biological markers that accurately identify PPD patients would significantly enhance both aspects.

Despite the extensive body of epidemiological research on PPD, comparatively few studies have systematically explored its biological determinants, underscoring the need for further investigation in this domain. In this context, our goal is to fill existing research gaps by outlining a consolidated model of the biological basis of postpartum depression. A unified model like this could advance more effective and targeted screening, help identify novel potential treatment targets, and explain differences in the probability, onset, and gravity of this condition. In this study, we aimed to investigate the biological pathways that may collaborate to heighten the risk of postpartum depression. Therefore, we assessed the potential links between biological markers in the blood and measured depressive symptomatology by EPDS score in postpartum women. The goal was to determine how blood levels of pregnancy-related hormones (estrogen and progesterone), vitamin D, markers of inflammatory response (white blood cell count, C-reactive protein, fibrinogen, neutrophil count, lymphocyte count, and ferritin), indicators of anemia (hemoglobin, red blood cell count, hematocrit, and ferritin), thyroid hormones (TSH, FT3, and FT4) and markers of coagulation abnormalities (D-dimer, platelets, fibrinogen, APTT, and INR) are associated with increased EPDS scores for postpartum depression. More research is needed to understand these relationships and their clinical implications fully.

## 2. Materials and Methods

### 2.1. Study Design and Sample Description

Our research was designed as a cross-sectional analysis of postpartum women, which was conducted in our tertiary academic public hospital (Obstetrics and Gynecology Clinical Section I—Timis County Emergency Clinical Hospital “Pius Brînzeu” from Timisoara, Romania) between 2023 and 2025, with the primary purpose of exploring the biological pathways that may interact to increase the risk for postpartum depression while explaining the variability in the likelihood and occurrence of this condition.

The study involved a sample of 860 mothers who had expressed interest in this subject. Women were invited to be enrolled in our study during the first 1–2 weeks postpartum, on the day of hospital discharge. For the purposes of our investigation, participants were thoroughly investigated and selected according to a comprehensive group of criteria for participant selection and exclusion to ensure a specific, valid, and consistent sample of participants. Overall, 860 mothers were selected and enrolled as eligible subjects for this research.

#### Criteria for Inclusion and Exclusion

Participants were approved for enrollment in the study if they matched the criteria listed below:

- Postpartum women aged between 18 and 50 years, who were hospitalized in our clinic;
- Women who gave birth in the previous 1–2 weeks ahead of survey completion;
- No earlier records of psychiatric disorders;
- No history of postpartum depression in previous pregnancies;
- Postpartum women who expressed enthusiasm for the subject and authorized their participation after receiving study details.

Participants were omitted from the research if they fulfilled the below criteria for exclusion:

- Women with pregnancies classified as high risk, with cases such as pregnancy-induced hypertension, preeclampsia, eclampsia, pregnancy-associated diabetes mellitus, intrahepatic cholestasis of pregnancy, intrauterine growth restriction, symptomatic maternal uretero-hydronephrosis in pregnancy, chronic illnesses, Rh incompatibility, chromosomal abnormalities, or other known fetal anomalies;
- Women who have experienced previous psychiatric conditions or mental health challenges;
- Women who have been exposed to psychotropic drug treatments in the past or are currently using them;
- Women diagnosed with mental health disorders or experiencing substance abuse issues, including those related to drugs or alcohol;
- Women with a prior history of blood coagulation abnormalities or any thromboembolic disease.
- Women who had negative experiences and suffered adverse reactions following prior peripheral blood collection procedures;
- Women identified as having infectious illnesses such as hepatitis B/C, HIV, or AIDS.

Informed consent was previously obtained, individually, from each participant, before the initiation of the study, as it involved a group of participants with increased vulnerability and the collection of sensitive data.

### 2.2. Data Collection Methods

Postpartum depressive disorder is a condition in which psychometric assessment is essential for screening and confirming the diagnosis. In accordance with the 2014 National Institute for Health and Care Excellence guidelines on antenatal and postnatal mental health [22], all postpartum women completed the Edinburgh Postnatal Depression Scale questionnaire, which served as a screening instrument for clinical signs of postpartum depression during the first 1–2 weeks after childbirth, on the day of hospital discharge.

The baseline characteristics of postpartum mothers were collected from electronic hospital databases and handwritten patient files, which were evaluated in detail by qualified clinicians engaged in the study. The medical histories of the patients were documented, with a focus on psychiatric-related disorders. To develop a clearer understanding of postpartum depression and the determinants shaping its development in the study participants, the database included extensive data covering the following parameters: age, legal relationship status, background, completed education level, work environment and professional exposure risks, level of socio-economic well-being, health status, history of personal health conditions, parity, reproductive method leading to conception, type of delivery, number of miscarriages, and number of elective abortions. Peripheral blood samples were collected from all 860 eligible participants on the day of hospital discharge. Venipuncture was performed as part of standard clinical procedures at discharge, and blood samples were collected into gel separator tubes (BD Vacutainer^®^, Becton, Dickinson and Company, Franklin Lakes, NJ, USA) designed for serum collection. Venous blood samples were collected via venipuncture into evacuated tubes with color-coded stoppers: lavender (EDTA) for hematological analyses, red (without anticoagulant) for serum collection, and blue (citrate) for coagulation studies. The blood samples were processed and analyzed in an accredited laboratory (the Clinical Laboratory of the “Pius Brinzeu” Emergency Clinical County Hospital in Timisoara) using standardized protocols. Complete blood cell count analysis was performed on whole blood samples. Coagulation parameters were assessed using plasma, while all other biochemical analyses were conducted using serum. Samples for biochemical and coagulation studies were centrifuged at 2500 revolutions per minute (rpm) for 10 min. Complete blood count (CBC) analyses were performed using the Nikon MEK-9100 analyzer, based on the principle of flow cytometry. Biochemical parameters were measured on the ADVIA Centaur XPT system (Siemens Healthineers, Erlangen, Germany), employing chemiluminescence-based detection. Coagulation assays were conducted using the ACL TOP 750 analyzer (Instrumentation Laboratory, Bedford, MA, USA). Blood levels of pregnancy-related hormones (estrogen and progesterone), vitamin D, biochemical markers of inflammatory response (white blood cell count, C-reactive protein, fibrinogen, neutrophil count, lymphocyte count, and ferritin), anemia-related biomarkers (hemoglobin, red blood cell count, hematocrit, and ferritin), thyroid hormones (TSH, FT3, and FT4) and markers of coagulation abnormalities (D-dimer, platelets, fibrinogen, APTT, and INR) were evaluated. All biomarker measurements were conducted on the day of hospital discharge and subsequently compared with the corresponding normal reference value ranges for each pregnancy-related hormone at 1–2 weeks postpartum: estradiol (<100 pg/mL), progesterone (<1 ng/mL or 1–3 ng/mL); vitamin D (30–100 ng/mL); C-reactive protein (0–10 mg/L); white blood cell count (4000–9500/μL); neutrophil count (1800–6700/μL); lymphocyte count (800–3800/μL); fibrinogen (200–393 mg/dL); ferritin (4.5–170 μg/L); hemoglobin (11.5–15 g/dL); hematocrit (35–46%); red blood cell count (4,000,000–5,500,000/μL); TSH (0.55–4.78 mIU/L); FT3 (3.54–6.47 pmol/L); FT4 (11.5–22.7 pmol/L); D-dimer (0–243 ng/mL); platelets (150,000–400,000/μL); INR (0.80–1.07 INR); and APTT (25.1–36.5 s). It is essential to note that this process took place in a specialized medical laboratory setting, where qualified clinicians evaluated the results and reviewed the admission details in conjunction with the patient’s personal care needs. These results provided a complete picture of the patient’s health status at the time point of hospital discharge, giving healthcare professionals important details to analyze potential threats and ensure appropriate medical recommendations.

### 2.3. Edinburgh Postnatal Depression Scale Questionnaire

The American College of Obstetricians and Gynecologists (ACOG), the American Academy of Family Physicians (AAFP), and, also, the American Academy of Pediatrics (AAP) all advocate for screening all postpartum mothers for perinatal depression by applying the EPDS questionnaire [23]. Perinatal depression screening should be consistently integrated into prenatal and postpartum care routines, using tools like the EPDS questionnaire to detect individuals at elevated risk [24]. Several screening tools are available, including the Patient Health Questionnaire-9 (PHQ-9) and the Generalized Anxiety Disorder 7-item Scale (GAD-7). However, the most widely adopted assessment tool is the EPDS questionnaire that takes only a few minutes for patients to complete [25,26]. The EPDS has been confirmed as a reliable tool for clinical diagnosis in more than 37 languages, extending across global populations and regions, including Romania [27,28,29].

The EPDS is a 10-item self-report scale, commonly used in perinatal mental health research [30,31], designed to assess the severity of depression symptoms experienced by the woman over the past seven days. It is an efficient and effective tool for the early identification of women at risk for maternal perinatal depression [32,33]. Scores exceeding 10 suggest possible depression (minor depressive disorder), while scores equal to or exceeding 13 suggest the presence of major depression (moderate to severe) [34].

The psychometric properties in primary healthcare were as follows: 86% sensitivity (correctly identifying actual cases), 78% specificity (correctly identifying individuals without the condition), and 73% positive predictive value (the proportion of respondents who scored positively on the test and were subsequently diagnosed with a mental disorder through clinical interview) [25,35]. The widespread use of this brief instrument can be attributed to the original British validation study [25], where nine out of ten women diagnosed with postpartum depression by a psychiatrist were accurately identified through a blinded comparison, with scores exceeding a designated cut-off on the EPDS [35].

### 2.4. Statistical Analysis

Data analysis was performed using JASP v0.19.3 statistical software [36], a high-level instrument used in statistical analysis in the biomedical field. Quantitative variables were expressed as mean ± standard deviation or as median with interquartile range, as appropriate.

The Kruskal–Wallis test was utilized to assess differences among more than two independent numerical groups, followed by pairwise evaluations of groups using the Mann–Whitney U test to achieve a more nuanced analysis. Furthermore, a multivariate linear regression analysis was conducted to identify the variables independently associated with the EPDS score. Spearman’s rank correlation analysis was applied to evaluate the strength and direction of the correlations between relevant variables. Statistical significance was defined by a *p*-value cutoff of 0.05. A correlation analysis was performed to determine and assess the relationship between pre-existing medical conditions and the occurrence of postpartum depression. To underscore statistically significant relationships, correlation coefficients and related *p*-values were presented.

This investigative approach enabled a thorough and precise analysis of the data, providing valuable insights into the clinical and obstetric profiles of women with postpartum depression within the specific context of this medical framework.

### 2.5. Ethics Declarations

This study was carried out under the guidelines of the Declaration of Helsinki and consistent with the European Union General Data Protection Regulation (GDPR). It additionally received authorization from the appropriate Local Commission of Ethics for Scientific Research from the Timis County Emergency Clinical Hospital “Pius Brinzeu”, Timisoara, Romania, with approval reference number 422/04.12.2023.

The medical records of participants were stored in a secure database following all required guidelines, with relevant privacy regulations, and access to these records was granted only after an informed consent form was completed and signed by the patient. Following ethical guidelines, all participants gave their written informed consent before participating in the research. This included their consent to participate in the survey actively, consent for collecting peripheral blood samples for analysis, and authorization to obtain their personal and health-related information. Additionally, this study implemented further measures to ensure the protection of personal and confidential information for all study subjects. Complete anonymization was applied to all collected data, guaranteeing that no personal identifiers were linked to the information. This rigorous approach further highlighted this study’s dedication to upholding the highest ethical standards throughout its duration.

## 3. Results

A total of 860 mothers were individually surveyed and completed the Edinburgh Postnatal Depression Scale (EPDS) questionnaire during the first 1–2 weeks postpartum, on the day of hospital discharge. A cutoff score of ≥13 was used to identify major postpartum depressive disorder, while a score > 10, but <13, indicated possible depression (minor depressive disorder). In total, 10.12% (87 mothers) were identified as having major depressive disorder, 10.93% (94 mothers) were clinically diagnosed with minor depressive disorder, and 78.95% (679 mothers) did not show any symptoms indicative of depressive disorder.

The analysis represented nominal data using numerical counts and corresponding percentages (n, %), while the presentation of numerical data included mean values, standard deviations, and medians, along with interquartile ranges.

The blood levels of pregnancy-related hormones (estrogen and progesterone), vitamin D, biochemical markers of inflammatory response (white blood cell count, C-reactive protein, fibrinogen, neutrophil count, lymphocyte count, and ferritin), indicators of anemia (hemoglobin, red blood cell count, hematocrit, and ferritin), thyroid hormones (TSH, FT3, and FT4) and markers of coagulation abnormalities (D-dimer, platelets, fibrinogen, APTT, and INR) were evaluated based on the presence or absence of postpartum depression (Table 1).

Table 1 lists the results obtained after the Kruskal–Wallis test. The blood levels of pregnancy-related hormones (estrogen and progesterone), thyroid hormones (TSH, FT3, and FT4), biochemical markers of inflammatory response (C-reactive protein and ferritin), vitamin D, and markers of coagulation abnormalities (APTT and INR) were significantly associated with the occurrence of postpartum depression.

**Figure 1:**
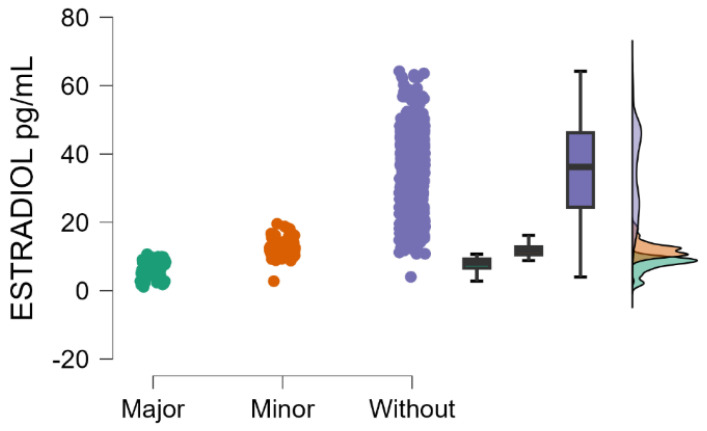
Blood levels of estradiol depending on the severity of postpartum depression.

Estradiol blood levels decrease significantly with the increasing severity of postpartum depression (Kruskal–Wallis’s test, *p* < 0.001; Dunn’s Post Hoc Comparison—Table 2 and Figure 1).

**Figure 2:**
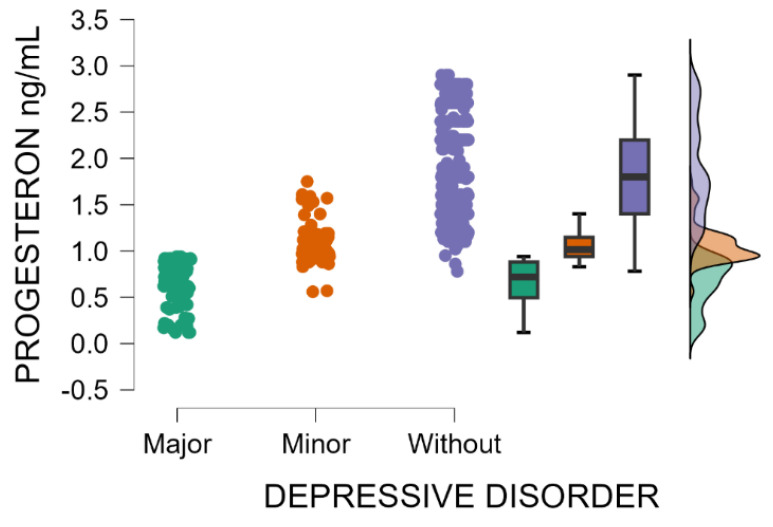
Blood levels of progesterone depending on the severity of postpartum depression.

Likewise, progesterone blood levels decrease significantly with the increasing severity of postpartum depression (Kruskal–Wallis’s test, *p* < 0.001; Dunn’s Post Hoc Comparison—Table 3 and Figure 2). After childbirth, the endocrine system undergoes marked fluctuations in hormone concentrations, particularly reproductive hormones, shown to be associated with the initiation, development, and intensity of postpartum depressive disorder.

**Figure 3:**
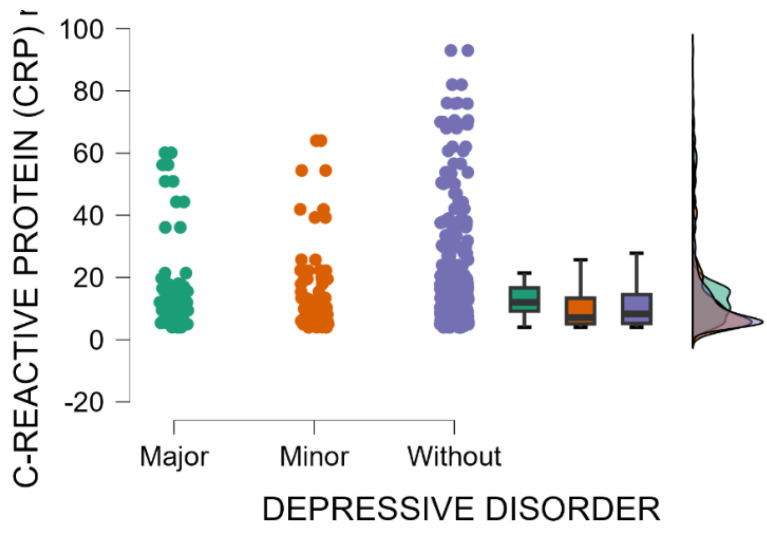
Blood levels of C-reactive protein depending on the severity of postpartum depression.

Blood levels of C-reactive protein are significantly higher in mothers with major postpartum depression compared with those with minor postpartum depressive disorder or without depression (Kruskal–Wallis’s test, *p* = 0.001; Dunn’s Post Hoc Comparison—Table 4 and Figure 3). The immune shift following delivery may trigger a temporary increase in inflammation in susceptible individuals, with elevated levels of inflammatory markers, such as C-reactive protein (CRP), potentially indicating a connection between the immune response and postpartum depression [37].

**Figure 4:**
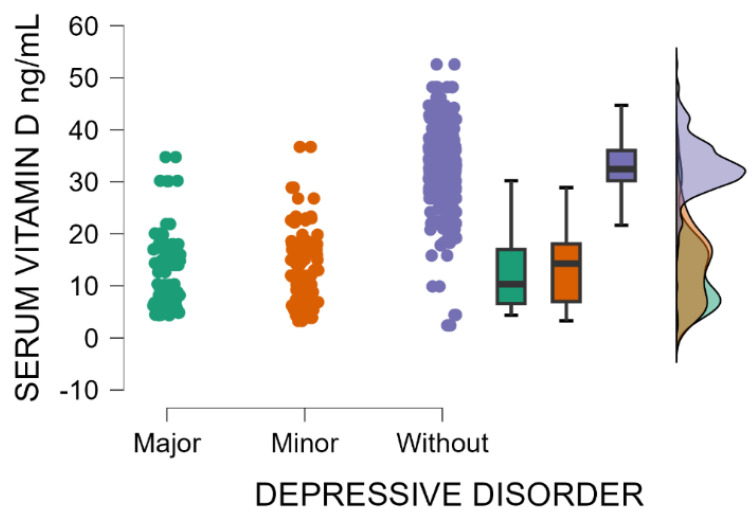
Blood levels of vitamin D depending on the severity of postpartum depression.

Patients without postpartum depression have significantly higher blood levels of vitamin D compared with those with minor or major postpartum depressive disorder (Kruskal–Wallis’s test, *p* < 0.001; Dunn’s Post Hoc Comparison—Table 5 and Figure 4). Vitamin D deficiency may be implicated as a risk factor for postpartum depression.

**Figure 5:**
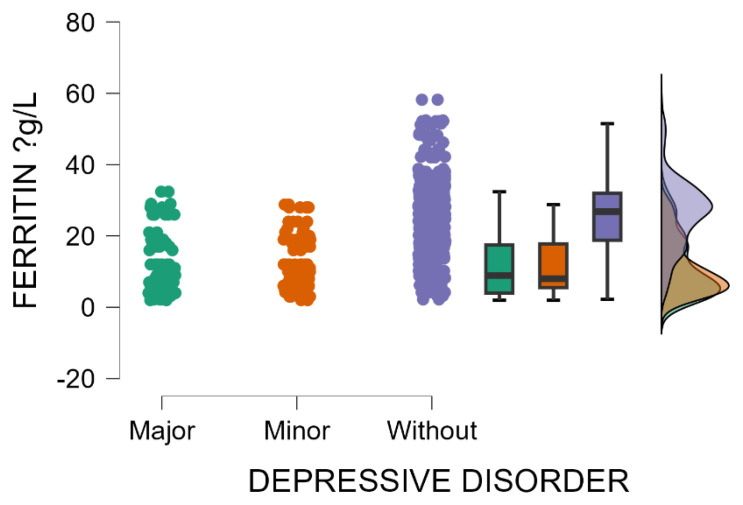
Blood levels of ferritin depending on the severity of postpartum depression.

Patients without postpartum depression have significantly elevated blood levels of ferritin compared with those with minor or major postpartum depressive disorder (Kruskal–Wallis’s test, *p* < 0.001; Dunn’s Post Hoc Comparison—Table 6 and Figure 5). These results suggest that mothers with low ferritin levels during the postpartum period are potentially more susceptible to depression. The link between postpartum depression and ferritin levels is an emerging area of research, with some evidence indicating that low ferritin (low iron stores) may be involved in triggering or aggravating peripartum depressive disorder.

A significant association was observed between maternal postpartum depression and blood levels of APTT (Kruskal–Wallis’s test, *p* = 0.021). Mothers with an EPDS score indicating major postpartum depression have significantly higher blood levels of APTT compared with those without postpartum depressive disorder (Dunn’s Post Hoc Comparison—Table 7, *p* = 0.006).

Additionally, it is essential to note a notable association observed between maternal postpartum depression and INR blood levels (Kruskal–Wallis’s test, *p* = 0.041). Mothers with an EPDS score indicating major postpartum depression have significantly elevated blood levels of INR compared with those with minor postpartum depressive disorder or without depression (Dunn’s Post Hoc Comparison—Table 8).

**Figure 6:**
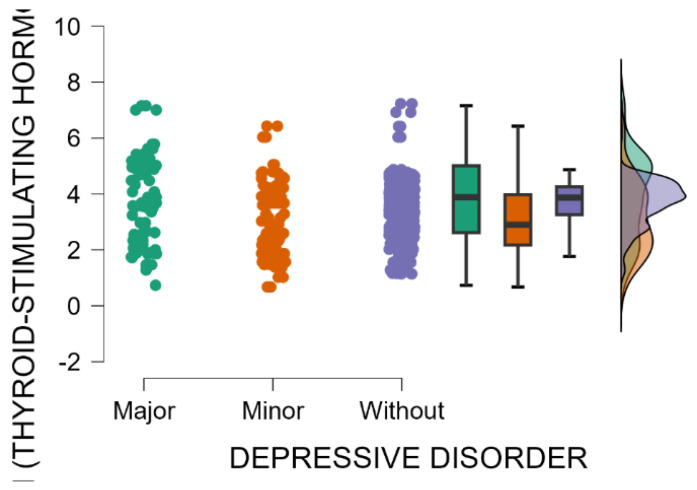
Blood levels of TSH depending on the severity of postpartum depression.

A significant association was observed between the occurrence of maternal postpartum depression and blood levels of thyroid-stimulating hormone (Kruskal–Wallis’s test, *p* < 0.001). Mothers with an EPDS score indicating major postpartum depression have significantly higher blood levels of TSH compared with those with minor postpartum depressive disorder or without depression (Dunn’s Post Hoc Comparison—Table 9 and Figure 6).

**Figure 7:**
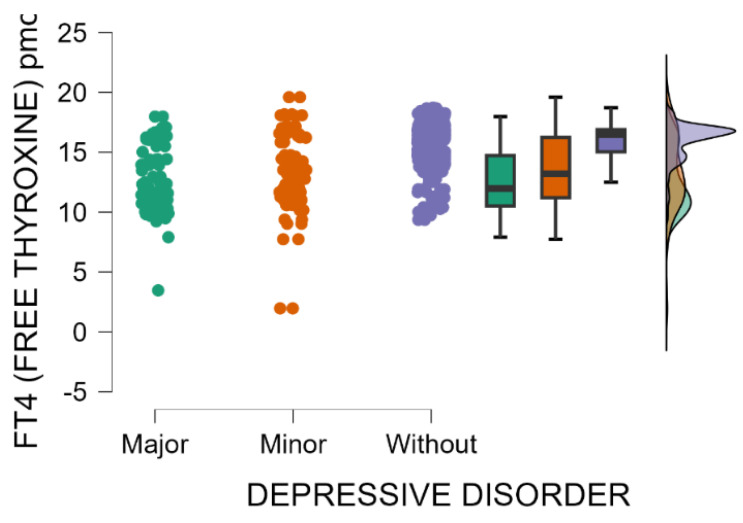
Blood levels of FT4 depending on the severity of postpartum depression.

The blood levels of FT4 significantly decrease as postpartum depression becomes more severe (Kruskal–Wallis’s test, *p* < 0.001; Dunn’s Post Hoc Comparison—Table 10 and Figure 7).

**Figure 8:**
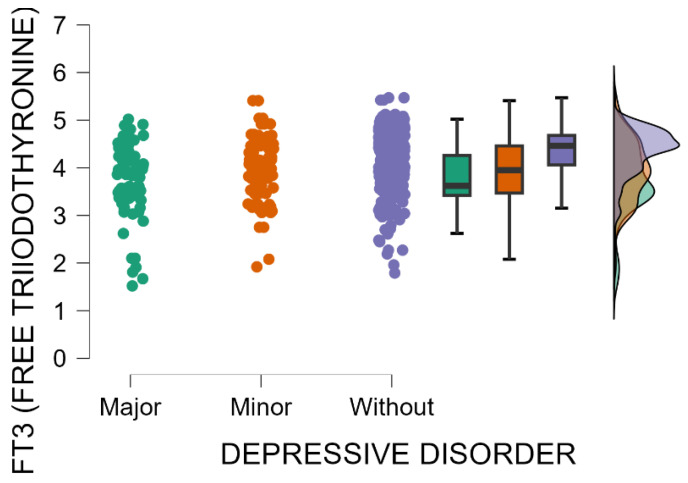
Blood levels of FT3 depending on the severity of postpartum depression.

The blood levels of FT3 significantly decrease as postpartum depression becomes more severe (Kruskal–Wallis’s test, *p* < 0.001; Dunn’s Post Hoc Comparison—Table 11 and Figure 8).

The fluctuation in thyroid hormone levels during the postpartum period may contribute to mood disturbances and elevate the risk of developing postpartum depression. Elevated thyroid-stimulating hormone (TSH) levels coupled with low thyroid hormone levels (FT3 and FT4) typically indicate hypothyroidism (underactive thyroid). Hypothyroidism appears to be the most common way in which thyroid dysfunction is associated with postpartum depression.

A multivariate linear regression analysis was conducted, with the EPDS score designated as the dependent variable and the remaining continuous variables derived from the laboratory analyses serving as independent predictors. The model demonstrated a statistically significant association (*p* < 0.001), indicating a meaningful relationship between the laboratory parameters and EPDS scores (Table 12).

The regression model identified the following variables as significant predictors of elevated Edinburgh Postnatal Depression Scale (EPDS) scores: increased neutrophil count, decreased serum vitamin D levels, prolonged activated partial thromboplastin time (APTT), reduced serum ferritin levels, decreased concentrations of estradiol and progesterone, and elevated thyroid-stimulating hormone (TSH) levels.

In the regression model, negative coefficients indicate an inverse association, whereby lower values of the corresponding variable are associated with higher EPDS scores. Conversely, positive coefficients indicate a direct association, with higher values of the variable corresponding to increased EPDS scores.

Table 13 displays the results of the Spearman non-parametric correlation analysis between the EPDS score and each of the numerical laboratory variables included in the previously described regression model.

Low serum vitamin D levels were significantly and strongly correlated with higher EPDS scores (Spearman’s rho = −0.69, *p* < 0.001). Reduced serum vitamin D levels during or following birth may represent a potential risk factor for the development of postpartum depression.

APTT values showed a statistically significant, weak, and positive correlation with EPDS scores (Spearman’s rho = 0.104, *p* = 0.002). Prolonged activated partial thromboplastin time (APTT) serum values may constitute a contributing factor in the development of postpartum depression.

A statistically significant moderate inverse correlation was observed between serum ferritin levels and EPDS scores (Spearman’s rho = −0.473, *p* < 0.001). Low serum ferritin concentrations in the peripartum period may be implicated as a potential risk factor in the development of postpartum depression.

Statistically significant inverse correlations were observed between EPDS scores and several hormonal parameters. Serum estradiol levels showed an almost perfect negative correlation with EPDS scores (Spearman’s rho = −0.922, *p* < 0.001). Likewise, serum progesterone levels demonstrated a strong inverse correlation (Spearman’s rho = −0.863, *p* < 0.001). The abrupt decline in pregnancy-associated hormones, particularly estrogen and progesterone, both of which are markedly elevated during gestation, is well documented in the immediate postpartum period. This sudden hormonal shift is believed to contribute to postpartum mood disturbances and may play a role in the onset of postpartum depression. In the present analysis, the reduced concentrations of estrogen and progesterone in serum were strongly linked to elevated EPDS scores, suggesting a correlation between reduced hormonal concentrations and the increased severity of depressive symptoms.

Additionally, weaker but statistically significant negative correlations were found between EPDS scores and both serum free thyroxine (FT4) levels (Spearman’s rho = −0.306, *p* < 0.001) and free triiodothyronine (FT3) levels (Spearman’s rho = −0.216, *p* < 0.001). Reduced blood levels of free thyroxine (FT4) and free triiodothyronine (FT3) were significantly associated with higher EPDS scores, suggesting that thyroid hormone deficiency may serve as a potential risk factor for postpartum depression.

## 4. Discussion

This clinical research study involved a clinical cohort of women in the postpartum period with no prior diagnosis of major depression. The inclusion criterion, excluding individuals with a personal psychiatric history, was deliberately implemented to minimize the risk of confounding factors due to potential relapse episodes and to ensure that newly emergent depressive symptoms were not attributable to pre-existing psychiatric conditions. Overall, 860 postpartum mothers responded to the EPDS questionnaire within the initial 1–2 weeks post delivery, specifically on the day of hospital discharge, through individual survey administration. Of the total cohort, 10.12% (*n* = 87) met the diagnostic criteria for major depressive disorder, 10.93% (*n* = 94) were identified with minor depressive disorder, and 78.95% (*n* = 679) exhibited no clinical signs indicative of a depressive disorder.

The findings of this study indicate that the severity of postpartum depressive symptoms was associated with the serum concentrations of specific blood-based biomarkers measured in postpartum samples. The serum concentrations of pregnancy-related hormones (estrogen and progesterone), vitamin D, inflammatory biomarkers (C-reactive protein, fibrinogen, white blood cell count, neutrophil count, lymphocyte count, and ferritin), hematological parameters indicative of anemia (hemoglobin, hematocrit, red blood cell count, and ferritin), thyroid hormones (TSH, FT3, and FT4), coagulation markers (D-dimer, platelet count, fibrinogen, APTT, and INR) were evaluated with the presence of postpartum depression. Among these, significant associations with postpartum depression were observed for the serum levels of pregnancy-related hormones (estrogen and progesterone), thyroid hormones (TSH, FT3, and FT4), inflammatory markers (C-reactive protein and ferritin), vitamin D, and coagulation parameters (APTT and INR).

These findings align with and extend the existing literature suggesting that the abrupt hormonal fluctuations following childbirth, particularly in estrogen and progesterone levels, may play a pivotal role in the pathophysiology of postpartum depression. The peripartum period constitutes a critical window in which women are at increased risk of developing depressive episodes, mainly attributable to significant fluctuations in sex hormone levels. Given the complex physiological changes that characterize normal pregnancy and the postpartum period, investigating the endocrine underpinnings of postpartum depression presents substantial methodological challenges. Rigorous study designs necessitate adequately powered sample sizes and the precise timing of biological assessments across defined phases of the postpartum period.

The rapid decline in estradiol and progesterone levels following parturition has understandably drawn considerable attention in postpartum depression research. Given the profound neuroendocrine shifts occurring during this period, there is a growing interest in these reproductive hormones, which are thought to be key contributors to the underlying mechanisms of postpartum depression.

It is well established that estrogen blood levels rise significantly throughout each trimester of pregnancy, particularly experiencing a 100- to 1000-fold increase in the last trimester, primarily due to placental synthesis. This may explain the particular vulnerability of the endocrine profile during pregnancy to the effects of abrupt estrogen withdrawal. Following delivery, these levels decline abruptly, reaching baseline pre-follicular levels around the fifth day after childbirth [38]. Mood disturbances, including symptoms of depression, anxiety or irritability, are also commonly observed during menstruation and menopause, which are characterized by significant declines in circulating sex hormone levels [39]. Due to these hormonal fluctuations, numerous studies have identified a post-delivery state of “estrogen withdrawal”, which has been implicated in the development of postpartum depression. This represents the most extensively investigated biological model for the pathogenesis of postpartum depression. Several experimental studies have demonstrated that following delivery—specifically after estradiol withdrawal—subjects exhibit increased behavioral despair and depressive symptoms, supporting the role of estrogen decline in the etiology of postpartum mood disturbances [40,41]. Hypoestrogenic states at other stages of a woman’s life, such as during the perimenopausal transition, have been associated with diminished psychological well-being. Notably, estradiol therapy has demonstrated potential therapeutic benefits in women experiencing perimenopausal depression, suggesting a modulatory role of estrogen in mood regulation [42]. Moreover, treatment with estradiol at relatively high doses has been shown to exert therapeutic effects in women with postpartum depression [43,44], further supporting the involvement of estrogen in the disorder’s pathophysiology. The release of several hormones and neurotransmitters has been demonstrated to be triggered by estradiol, including serotonin, dopamine, GABA, norepinephrine, and corticosterone, playing an important role in emotional regulation. Following childbirth, the abrupt decline in estrogen levels may lead to a reduction in neurotransmitter availability or alterations in the density or affinity of monoamine receptors, thereby contributing to the onset of mood disturbances [45].

Progesterone plays a crucial role in facilitating uterine preparation for pregnancy and maintaining its progression. Throughout the early weeks of pregnancy, the corpus luteum is the main source of progesterone production, as the placenta progressively takes over this role between the seventh and ninth week of pregnancy [39]. Just prior to delivery, progesterone levels rise significantly, reaching concentrations of 200–2000 nmol/L, but following childbirth, these levels decline rapidly; nearly 50% of circulating progesterone is removed from the bloodstream during the initial 30 min, and its levels normalize to those typical of the luteal phase within 2 to 3 h [39]. The rapid decrease in progesterone levels has been referred to as a “withdrawal state”, analogous to the “estrogen withdrawal” observed after delivery. Although antidepressants are considered the first-line treatment for depression, research has explored the potential role of hormonal treatments as adjunctive therapy for women diagnosed with postpartum depression, in light of the pronounced hormonal changes associated with pregnancy. In the United States, two phase 3 studies were carried out using a double-blind, randomized, placebo-controlled methodology and assessed the therapeutic value of brexanolone in women with postpartum depression [46]. Brexanolone is an injectable form of allopregnanolone, a metabolite of progesterone, known to influence synaptic as well as extrasynaptic GABA-A receptor activity [46]. The participants were allocated at random to receive either brexanolone dosed at 90 mg/kg/h, brexanolone dosed at 60 mg/kg/h, or a placebo, administered intravenously over 60 h in the first study, while the second study involved the administration of either brexanolone dosed at 90 mg/kg/h or a placebo for the same duration [46]. The results of the first study demonstrated that brexanolone significantly outperformed the placebo, demonstrating a significant variation in efficacy between brexanolone dosed at 90 mg/kg/h and 60 mg/kg/h [46]. The therapeutic effect was observed to begin within 60 h, with improvements that persisted for up to 30 days post treatment. Notably, 94% of the women who responded to brexanolone experienced no relapse within 30 days [46]. The U.S. Food and Drug Administration subsequently approved brexanolone intravenous infusion therapy in March 2019 as a first-line therapy explicitly for postpartum depression [47].

As evidenced by the results of our study, estradiol and progesterone blood levels decrease significantly with the increasing severity of postpartum depression. There is a well-established connection between endocrine withdrawal occurring post delivery and the beginning of postpartum depressive symptoms. This could be particularly prevalent in women with a history of the condition. Additionally, genetic factors may contribute to individual susceptibility to the abrupt declines in hormone blood levels. It is important to note that even among women previously affected by postpartum depression, the responses to hormonal withdrawal can vary between pregnancies [39]. Currently, intravenous brexanolone infusion remains the only treatment specifically approved by the FDA for postpartum depression. This therapy represents a significant advancement, offering the potential for a sustained therapeutic response and reducing the overall burden for women affected by this condition [39,47].

Furthermore, the dysregulation of thyroid function and systemic inflammation, as evidenced by altered thyroid hormone levels and elevated concentrations of C-reactive protein, may play a contributory role in the neuroendocrine and immunological mechanisms underlying mood disorders.

In our study, we detected a statistically significant relationship involving maternal postpartum depression and alterations in thyroid function, particularly in TSH and thyroid hormones FT3 and FT4. Mothers with an EPDS score indicative of major PPD had significantly higher TSH levels compared with those with minor PPD or no depressive symptoms. Additionally, as the severity of postpartum depression increased, blood levels of FT4 and FT3 significantly decreased. These findings are consistent with the existing literature that highlights the role of thyroid dysfunction in the development and progression of postpartum depression. Thyroid hormone fluctuations during the postpartum period are a well-documented phenomenon, with recent studies suggesting that altered thyroid function may contribute to mood disturbances. Elevated TSH levels coupled with lower FT3 and FT4 levels typically suggest hypothyroidism, a condition most commonly linked with mood disorders, including PPD. A study conducted in Sweden found that elevated TSH levels were associated with a higher risk of developing depressive symptoms postpartum, with hypothyroidism being a significant risk factor [48]. Serum TSH measurement is a diagnostically sensitive and cost-effective test commonly employed to detect both hyperthyroidism and hypothyroidism [48]. In conjunction with FT4, it is instrumental in differentiating between subclinical and overt hypothyroidism, as well as in assessing treatment efficacy [48]. Furthermore, a systematic review has shown that low FT3 and FT4 levels, even within the normal reference range, are associated with higher postpartum mood disturbance scores [49]. The fluctuation in thyroid hormone levels, particularly the lowering of FT3 and FT4, as PPD becomes more severe, underscores the potential role of thyroid dysfunction in the pathophysiology of postpartum depression. The findings of our study suggest that elevated TSH levels coupled with low FT4 levels typically indicate hypothyroidism and could serve as a valuable screening tool for identifying individuals at elevated risk for PPD, thereby providing a basis for further psychiatric evaluation and intervention. Further studies are needed to explore the biological pathways associating thyroid dysfunction with PPD, and whether early interventions aimed at correcting thyroid imbalances could serve as a preventive measure for postpartum depression.

Consistent with the growing evidence linking systemic inflammation to mood disorders, our findings demonstrate that blood levels of C-reactive protein (CRP) are significantly elevated in patients diagnosed with major postpartum depression when compared with individuals presenting with minor postpartum depressive symptoms or no depressive symptoms at all. This observation supports the hypothesis that inflammatory processes may play a more prominent role in the pathophysiology of major depressive episodes in the postpartum period [50]. A systematic review highlighted that elevated CRP levels at the end of pregnancy or immediately after delivery could predict postpartum depression (PPD). The review suggests that interactions between inflammation and the corticotropic axis might explain the onset of PPD, and epigenetic mechanisms could lead to a pro-inflammatory state [51]. Additionally, research indicates that the postpartum period is characterized by an increase in pro-inflammatory cytokines, likely induced by the physical trauma and labor associated with delivery. Pro-inflammatory cytokine levels remain elevated for at least the initial 72 h following delivery and could be associated with depressive symptoms [52]. Another study’s outcomes show that elevated serum levels of high-sensitivity C-reactive protein (Hs-CRP) and interleukin-6 (IL-6) following delivery are positively associated with EPDS scores ≥12, a threshold indicative of increased risk for postpartum depression. These inflammatory markers, measured shortly after childbirth, may serve as significant biological indicators of vulnerability to PPD [53]. Importantly, this study establishes a firm correlation between elevated serum concentrations of Hs-CRP and IL-6 at the time of postpartum admission and the occurrence of clinically significant depressive symptoms in the half-year following childbirth [53]. These results support the growing body of evidence implicating systemic inflammation in the etiology of postpartum mood disorders and highlight the potential utility of C-reactive protein as a predictive biomarker for early identification and intervention.

The assertion that patients without postpartum depression have significantly higher blood levels of vitamin D compared with those with minor or major PPD, and that vitamin D deficiency may be implicated as a risk factor for PPD, is supported by several studies. A study conducted in Iran found that women with PPD had significantly lower mean serum vitamin D levels (16.89 ± 7.05 ng/mL) compared with women without PPD (21.28 ± 7.13 ng/mL) [54]. Additionally, women with vitamin D levels below 20 ng/mL were 3.3 times more likely to have PPD than those with higher levels [54]. Another study from Australia observed that women in the lowest quartile for 25-hydroxyvitamin D status during pregnancy were more likely to report higher levels of postnatal depression symptoms at three days post delivery compared with women in the highest quartile [55]. The observed association between low vitamin D levels and depressive symptomatology, as previously reported in both general and perinatal populations, further underscores its potential significance as a modifiable risk factor in the context of mood disorders, highlighting the importance of monitoring and potentially correcting vitamin D levels during pregnancy and the postpartum period.

Several studies support the observation that women without postpartum depression (PPD) exhibit significantly higher serum ferritin levels compared with those diagnosed with minor or primary forms of PPD. These findings from our research suggest that low ferritin concentrations during the postpartum period may be associated with an increased susceptibility to depressive symptoms, highlighting iron status as a potential biological contributor to the development of PPD. A prospective cohort study conducted in Spain found that women who developed PPD had lower ferritin concentrations 48 h after delivery compared with those who did not develop PPD. Specifically, the mean ferritin levels were 15.4 μg/L in the PPD group versus 21.6 μg/L in the non-PPD group [56]. The investigation reported a 3.73 odds ratio related to postpartum depression in women with ferritin levels below 7.26 μg/L, indicating a strong significantly association between low ferritin blood levels and the development of PPD [56]. A literature review encompassing 17 studies also supports the link between low ferritin levels and the positive predictive value (PPV) of disease. A majority of studies (8 out of 10) reported an increased risk of postpartum depression in anemic women, with odds ratios ranging from 1.70 to 4.64 [57]. The review highlighted that low ferritin levels in the postpartum period, but not during pregnancy, were associated with an increased risk of PPD [57]. These findings collectively suggest that mothers with low ferritin levels during the postpartum period may exhibit a greater susceptibility to depressive disorders. The connection between ferritin levels and PPD is an emerging area of research, warranting further investigation to understand the underlying mechanisms and potential interventions better.

A noteworthy observation in our findings is the apparent association between maternal postpartum depression and altered coagulation parameters, particularly elevated levels of APTT and INR. Mothers with EPDS scores indicative of major postpartum depression demonstrated significantly higher APTT and INR levels compared with those with minor or no depressive symptoms. Although the current literature does not extensively document these specific associations, this novel finding suggests a plausible association between coagulation system dysregulation and biological pathways linked to PPD. To date, most research has focused on inflammatory markers, nutritional deficiencies, and hormonal changes as contributors to PPD, while the role of coagulation markers remains underexplored. The physiological changes following childbirth, including hemodynamic and hormonal fluctuations, could plausibly influence both mood regulation and coagulation pathways. The elevated APTT and INR observed in this study may reflect a state of subclinical inflammation characteristic of the postpartum period or endothelial dysfunction, which warrants further investigation. Future studies are needed to replicate these findings in larger, more diverse populations and to explore the underlying mechanisms that might link coagulation disturbances with depressive symptoms in the postpartum period. Clarifying this relationship could contribute to the development of novel biomarkers for the early identification of women at risk for PPD.

These findings highlight the multifactorial etiology of postpartum depression and emphasize the importance of incorporating biochemical and hormonal evaluations into early screening and preventative interventions.

This original research is characterized by some limitations that should be addressed. First, the scope of serum biomarker assessment was limited, as we did not include a broader panel of biological indicators known to be implicated in the pathophysiology of depression. Specifically, hormonal markers such as cortisol and allopregnanolone, which are critically involved in stress regulation and neuroendocrine adaptation during the peripartum period, were not assessed. Similarly, key pro-inflammatory cytokines, including interleukin-1β (IL-1β) and tumor necrosis factor-alpha (TNF-α), as well as neurotrophic factors like brain-derived neurotrophic factor (BDNF), were omitted. These markers may interact with high-sensitivity CRP and IL-6, potentially influencing both the onset and severity of postpartum depression (PPD).

Furthermore, the exclusion of genetic and epigenetic data, along with emerging exploratory biomarkers such as oxytocin and serotonin metabolites, limits our ability to capture the complex biological interplay underlying mood dysregulation in the postpartum period. The absence of these data may have contributed to an underestimation of particular biological vulnerabilities or masked relevant mechanistic pathways. Future studies incorporating a more comprehensive biomarker panel are warranted to elucidate the multifactorial nature of PPD and to enhance the predictive value of biological screening tools.

Second, the cross-sectional design of this study limits the ability to draw causal inferences from the observed associations. As an example, while elevated inflammatory marker (CRP) was associated with increased depressive symptoms, it remains unclear whether inflammation contributes to the onset of postpartum depression or whether depressive symptoms themselves induce an inflammatory response. The possibility of reverse causality, as well as the influence of unmeasured confounding variables, cannot be excluded. As such, the findings should be interpreted with caution. To establish temporal and potentially causal relationships between biomarkers and the development of postpartum depression, longitudinal studies with repeated measures across the peripartum period are warranted.

Third, a potential methodological limitation of this study is the reliance on the Edinburgh Postnatal Depression Scale (EPDS), a self-report screening instrument, rather than a structured clinical interview for the diagnosis of postpartum depression. While the EPDS is a widely validated and commonly used tool for identifying women at risk for PPD, it does not provide a definitive clinical diagnosis. The use of self-administered questionnaires may introduce reporting bias and limit diagnostic specificity, potentially leading to the misclassification of cases. Future studies would benefit from incorporating standardized psychiatric evaluations to confirm PPD diagnoses and enhance diagnostic accuracy.

Given the extensive number of biomarkers tested in relation to depressive symptoms, there is a heightened risk of Type I errors (false positives). This concern is particularly relevant in studies involving multiple hypothesis testing, where the probability of false positive findings increases. To address this issue, we employed the Post Hoc Dunn test following the Kruskal–Wallis’s test in our analysis of group differences based on depression severity. The Dunn test incorporates corrections for multiple comparisons, thereby reducing the likelihood of spurious associations. In future analyses, we intend to further assess the significance and clinical relevance of identified biomarkers by establishing specific thresholds and computing odds ratios (OR) along with corresponding 95% confidence intervals. This analytical strategy is expected to enhance the interpretability of the results, facilitate the identification of clinically meaningful biomarkers, and contribute to the development of objective, biologically based screening criteria for postpartum depression.

Finally, a key limitation of our study is its cross-sectional design, which precludes the ability to establish causal relationships between biomarker levels and postpartum depression (PPD). Since all measurements—including EPDS scores and blood biomarker levels—were collected at a single time point (1–2 weeks postpartum), it is not possible to ascertain whether changes in biomarker concentrations preceded the onset of depressive symptoms, occurred concurrently, or developed as a consequence. While elevated or lower levels of this biomarker were observed in a subset of individuals who subsequently developed postpartum depression (PPD), the cross-sectional nature of this measurement precludes conclusions regarding the temporal stability or trajectory of these biomarkers. It remains unclear whether the observed elevations persisted over time or whether the specific timing of measurement—immediately postpartum—was critical in predicting PPD onset. Longitudinal sampling across the peripartum and early postpartum periods is needed to clarify whether sustained or transient elevations or decreases in biomarker levels are more strongly associated with the development of PPD.

Upcoming research would benefit from the use of longitudinal approaches, encompassing the entire course of pregnancy and the postpartum period, with repeated assessments of depressive symptomatology alongside key biological variables. These should include reproductive hormones, vitamin D levels, inflammatory biomarkers, hematological indices relevant to anemia, thyroid function markers, and coagulation parameters. Such comprehensive analyses are warranted to elucidate the complex and potentially multifactorial pathophysiology of postpartum depression.

## 5. Conclusions

Biological theories regarding the pathophysiology of postpartum depression (PPD) share several similarities with those proposed for other psychiatric disorders. However, the postpartum period constitutes a distinct physiological and psychosocial state, characterized by unique endocrine fluctuations and life transitions that are unparalleled at any other stage in a woman’s life. Consequently, direct comparisons between peripartum depression and depressive episodes occurring outside the context of pregnancy and childbirth may not be entirely appropriate.

This observation contributes to a deeper understanding of the heightened vulnerability to depression in women during periods of significant hormonal fluctuations. These findings underscore the multifactorial nature of postpartum depression and highlight the relevance of integrating biochemical and hormonal assessments into early screening and preventive strategies. Our research focused on finding early biomarkers that might predict vulnerability to postpartum depression. These results support the growing body of evidence implicating them as predictive biomarkers for early identification and intervention. Should their predictive value be validated, the assessment of pregnancy-related hormones (estrogen and progesterone), thyroid function parameters (TSH, FT3, and FT4), inflammatory markers (C-reactive protein and ferritin), vitamin D levels, and coagulation indices (APTT and INR) could complement clinical evaluation to enable the early identification of individuals at elevated risk for peripartum depression. Further research incorporating a more comprehensive biomarker panel is warranted to test the value of these biomarkers in detecting individuals at heightened risk for postpartum depression and to explore potential adjunctive interventions beyond conventional antidepressant therapy. In such cases, targeted interventions, including vitamin D and iron supplementation, may be considered as part of a preventive strategy. Early interventions involving the monitoring of thyroid hormone levels during pregnancy and after birth, followed by the correction of any identified imbalances, may also be beneficial in preventing postpartum depression. Currently, the only treatment approved by the FDA is intravenous brexanolone infusion, indicated explicitly for postpartum depression [47]. These therapeutic approaches mark a significant advancement, offering the potential for sustained clinical improvement and alleviating the overall burden for women affected by this condition.

## Figures and Tables

**Table 1:** Blood levels of biological biomarkers depend on the occurrence of postpartum depression.

Variable	Depressive Disorder			*p* ^K-W^
	Major (*n* = 87)	Minor (*n* = 94)	Without (*n* = 679)	
Estradiol pg/mL	7.527 ± 2.322	12.086 ± 2.441	34.69 ± 12.23	<0.001 *
	8.2 (6.605–9.255)	11.935 (10.495–12.8)	36.2 (24.41–46.2)	
Progesterone ng/mL	0.653 ± 0.255	1.062 ± 0.201	1.876 ± 0.525	<0.001 *
	0.72 (0.495–0.88)	1.015 (0.94–1.147)	1.8 (1.4–2.2)	
C-Reactive Protein (CRP) mg/L	15.864 ± 13.333	12.911 ± 12.913	13.65 ± 13.543	0.001 *
	12 (9.2–16.65)	7.1 (5.05–13.35)	8.3 (5.2–14.5)	
Fibrinogen mg/dL	540.701 ± 107.327	536.979 ± 100.078	520.255 ± 101.291	0.051
	539 (461–615)	539 (472.5–585)	510 (450–573.5)	
Serum Vitamin D ng/mL	12.733 ± 7.217	13.913 ± 7.32	32.871 ± 5.847	<0.001 *
	10.33 (6.61–17)	14.29 (6.99–18.075)	32.44 (30.2–36)	
White Blood Cells (WBC/μL)	11,654.598 ± 3624.002	11,561.915 ± 3114.598	11,796.901 ± 8114.619	0.269
	10,990 (8910–14,000)	11,110 (9502.5–12,862.5)	10,610 (9010–12,905)	
Lymphocytes/μL	2321.609 ± 898.914	2250.851 ± 708.425	2319.131 ± 698.856	0.546
	2130 (1770–2670)	2140 (1800–2580)	2220 (1835–2750)	
Red Blood Cells (RBC/μL)	4.176 × 10^+6^ ± 623,947.836	4.224 × 10^+6^ ± 422,250.293	4.221 × 10^+6^ ± 406,882.265	0.933
	4.320 × 10^+6^ (3.830 × 10^+6^–4.625 × 10^+6^)	4.260 × 10^+6^ (3.968 × 10^+6^–4.478 × 10^+6^)	4.230 × 10^+6^ (3.940 × 10^+6^–4.480 × 10^+6^)	
Hemoglobin (Hb) g/dL	11.753 ± 1.65	12.163 ± 1.372	12.031 ± 1.198	0.195
	11.93 (10.95–12.79)	12.17 (11.242–13.313)	12.1 (11.25–12.86)	
Hematocrit (HCT) %	35.2 ± 5.079	36.496 ± 4.016	35.997 ± 3.413	0.163
	36.3 (32.4–38.85)	36.9 (33.575–39.575)	36.2 (34–38.35)	
Ferritin μg/L	11.591 ± 8.535	11.324 ± 7.547	25.879 ± 9.643	<0.001 *
	8.9 (4–17.5)	8 (5.5–17.75)	26.8 (18.8–32)	
Platelets (PLT/μL)	281,504.598 ± 95,854.625	268,565.957 ± 79,392.59	267,817.378 ± 78,995.401	0.588
	268,300 (214,150–329,300)	247,000 (217,275–301,625)	254,700 (210,800–313,800)	
D-Dimer ng/mL	775.575 ± 559.52	760.913 ± 485.908	806.439 ± 799.057	0.774
	615 (497–879)	678 (478.5–854.475)	618 (458–849)	
APTT s	28.744 ± 2.752	28.336 ± 2.652	28.101 ± 2.852	0.021 *
	28.9 (26.8–30.7)	28.3 (26.3–29.675)	27.8 (26.5–29.6)	
INR (INR)	0.964 ± 0.058	0.948 ± 0.048	0.951 ± 0.06	0.041 *
	0.96 (0.935–1)	0.95 (0.912–0.97)	0.95 (0.91–0.98)	
Neutrophils/μL	8398.046 ± 3579.134	8502.979 ± 2987.656	7888.999 ± 2638.115	0.076
	7100 (6170–9945)	8050 (6557.5–9287.5)	7530 (6130–9210)	
TSH (Thyroid-Stimulating Hormone) mIU/L	3.911 ± 1.498	3.095 ± 1.296	3.718 ± 0.83	<0.001 *
	3.885 (2.618–5.006)	2.897 (2.176–3.977)	3.87 (3.26–4.26)	
FT4 (Free Thyroxine) pmol/L	12.579 ± 2.749	13.305 ± 3.236	15.907 ± 1.631	<0.001 *
	11.97 (10.51–14.715)	13.19 (11.203–16.233)	16.46 (15.025–16.88)	
FT3 (Free Triiodothyronine) pmol/L	3.744 ± 0.749	3.923 ± 0.669	4.34 ± 0.531	<0.001 *
	3.62 (3.42–4.26)	3.95 (3.47–4.46)	4.46 (4.06–4.68)	

^K-W^—Kruskal–Wallis’s Test; *—significant difference.

**Table 2:** Dunn’s Post Hoc Comparison testing of estradiol blood levels depending on the severity of postpartum depression.

Dunn’s Post Hoc Comparison—Depressive Disorder—Estradiol					
Comparison	z	W_i_	W_j_	r_rb_	*p*
Major Minor	−2.597	47.615	143.559	0.942	0.009
Major Without	−16.677	47.615	519.283	0.997	<0.001
Minor Without	−13.746	143.559	519.283	0.966	<0.001

Note. Rank biserial correlation based on individual Mann–Whitney’s tests.

**Table 3:** Dunn’s Post Hoc Comparison testing of progesterone blood levels depending on the severity of postpartum depression.

Dunn’s Post Hoc Comparison—Depressive Disorder—Progesterone					
Comparison	z	W_i_	W_j_	r_rb_	*p*
Major Minor	−2.908	49.184	156.277	0.905	0.004
Major Without	−16.607	49.184	517.321	0.998	<0.001
Minor Without	−13.253	156.277	517.321	0.924	<0.001

Note. Rank biserial correlation based on individual Mann–Whitney’s tests.

**Table 4:** Dunn’s Post Hoc Comparison testing of C-reactive protein blood levels depending on the severity of postpartum depression.

Dunn’s Post Hoc Comparison—Depressive Disorder—PCR					
Comparison	z	W_i_	W_j_	r_rb_	*p*
Major Minor	3.15	522.241	406.223	0.247	0.002
Major Without	3.552	522.241	422.106	0.236	<0.001
Minor Without	−0.583	406.223	422.106	0.04	0.591

Note. Rank biserial correlation based on individual Mann–Whitney’s tests.

**Table 5:** Dunn’s Post Hoc Comparison testing of vitamin D blood levels depending on the severity of postpartum depression.

Dunn’s Post Hoc Comparison—Depressive Disorder—Vitamin D					
Comparison	z	W_i_	W_j_	r_rb_	*p*
Major Minor	−0.267	110.023	119.883	0.117	0.79
Major Without	−14.305	110.023	514.564	0.928	<0.001
Minor Without	−14.441	119.883	514.564	0.93	<0.001

Note. Rank biserial correlation based on individual Mann–Whitney’s tests.

**Table 6:** Dunn’s Post Hoc Comparison testing of ferritin blood levels depending on the severity of postpartum depression.

Dunn’s Post Hoc Comparison—Depressive Disorder—Ferritin					
Comparison	z	W_i_	W_j_	r_rb_	*p*
Major Minor	0.263	183.356	173.723	0.032	0.793
Major Without	−11.074	183.356	494.507	0.721	<0.001
Minor Without	−11.812	173.723	494.507	0.758	<0.001

Note. Rank biserial correlation based on individual Mann–Whitney’s tests.

**Table 7:** Dunn’s Post Hoc Comparison testing of APTT blood levels depending on the severity of postpartum depression.

Dunn’s Post Hoc Comparison—Depressive Disorder—APTT					
Comparison	z	W_i_	W_j_	r_rb_	*p*
Major Minor	1.636	498.874	438.415	0.142	0.102
Major Without	2.766	498.874	420.644	0.182	0.006
Minor Without	0.65	438.415	420.644	0.042	0.516

Note. Rank biserial correlation based on individual Mann–Whitney’s tests.

**Table 8:** Dunn’s Post Hoc Comparison testing of INR blood levels depending on the severity of postpartum depression.

Dunn’s Post Hoc Comparison—Depressive Disorder—INR					
Comparison	z	W_i_	W_j_	r_rb_	*p*
Major Minor	2.196	493.103	412.096	0.2	0.028
Major Without	2.412	493.103	425.027	0.157	0.016
Minor Without	−0.474	412.096	425.027	0.029	0.636

Note. Rank biserial correlation based on individual Mann–Whitney’s tests.

**Table 9:** Dunn’s Post Hoc Comparison testing of TSH blood levels depending on the severity of postpartum depression.

Dunn’s Post Hoc Comparison—Depressive Disorder—TSH					
Comparison	z	W_i_	W_j_	r_rb_	*p*
Major Minor	5.044	490.391	303.457	0.329	<0.001
Major Without	1.829	490.391	438.776	0.135	0.067
Minor Without	−4.915	303.457	438.776	0.329	<0.001

Note. Rank biserial correlation based on individual Mann–Whitney’s tests.

**Table 10:** Dunn’s Post Hoc Comparison testing of FT4 blood levels depending on the severity of postpartum depression.

Dunn’s Post Hoc Comparison—Depressive Disorder—FT4					
Comparison	z	W_i_	W_j_	r_rb_	*p*
Major Minor	−1.97	190.764	263.553	0.196	0.049
Major Without	−10.38	190.764	484.329	0.679	<0.001
Minor Without	−8.078	263.553	484.329	0.517	<0.001

Note. Rank biserial correlation based on individual Mann–Whitney’s tests.

**Table 11:** Dunn’s Post Hoc Comparison testing of FT3 blood levels depending on the severity of postpartum depression.

Dunn’s Post Hoc Comparison—Depressive Disorder—FT3					
Comparison	z	W_i_	W_j_	r_rb_	*p*
Major Minor	−1.184	256.098	299.83	0.113	0.237
Major Without	−7.597	256.098	470.936	0.498	<0.001
Minor Without	−6.261	299.83	470.936	0.399	<0.001

Note. Rank biserial correlation based on individual Mann–Whitney’s tests.

**Table 12:** Results of the linear regression model.

Independent Variables	Unstandardized	Standard Error	Standardized	t	*p*
Intercept	16.855	1.322		12.746	<0.001
WBC/μL	−6.646 × 10^−6^	7.726 × 10^−6^	−0.011	−0.86	0.39
RBC/μL	−6.858 × 10^−8^	1.915 × 10^−7^	−0.007	−0.358	0.72
Hb g/dL	−0.217	0.14	−0.06	−1.544	0.123
HCT %	0.046	0.055	0.037	0.845	0.398
PLT/μL	−9.348 × 10^−7^	7.353 × 10^−7^	−0.017	−1.271	0.204
Neutrophils/μL	7.003 × 10^−5^	2.104 × 10^−5^	0.043	3.328	<0.001
Lymphocytes/μL	−6.835 × 10^−5^	7.612 × 10^−5^	−0.011	−0.898	0.369
(CRP) mg/L	−0.008	0.005	−0.024	−1.843	0.066
Serum Vitamin D ng/mL	−0.086	0.009	−0.191	−9.68	<0.001
APTT s	0.092	0.02	0.057	4.662	<0.001
D-Dimer ng/mL	8.833 × 10^−5^	7.077 × 10^−5^	0.015	1.248	0.212
Fibrinogen mg/dL	5.518 × 10^−4^	5.554 × 10^−4^	0.012	0.994	0.321
INR	−1.557	0.968	−0.02	−1.608	0.108
Ferritin μg/L	−0.023	0.006	−0.056	−3.637	<0.001
Estradiol pg/mL	−0.135	0.006	−0.439	−20.8	<0.001
Progesterone ng/mL	−2.625	0.145	−0.366	−18.068	<0.001
TSH mIU/L	0.223	0.055	0.049	4.037	<0.001
FT4 pmol/L	0.004	0.03	0.002	0.144	0.886
FT3 pmol/L	−0.113	0.106	−0.015	−1.068	0.286

**Table 13:** Spearman’s correlation results with EPDS score.

Variable	Spearman’s Rho	*p*-Value
WBC/μL	0.018	0.591
RBC/μL	0.03	0.384
Hb g/dL	0.022	0.514
HCT %	0.03	0.377
PLT/μL	−0.033	0.328
Neutrophils/μL	0.028	0.418
Lymphocytes/μL	−0.018	0.598
(CRP) mg/L	0.02	0.557
Serum Vitamin D ng/mL	−0.69	<0.001
APTT s	0.104	0.002
D-Dimer ng/mL	0.032	0.35
Fibrinogen mg/dL	0.018	0.595
INR	0.047	0.172
Ferritin μg/L	−0.473	<0.001
Estradiol pg/mL	−0.922	<0.001
Progesterone ng/mL	−0.863	<0.001
TSH mIU/L	−0.035	0.301
FT4 pmol/L	−0.306	<0.001
FT3 pmol/L	−0.216	<0.001

## Data Availability

The original contributions presented in the study are included in the article; further inquiries can be directed to the corresponding author.

## Abbreviations

PPD	Postpartum depression
EPDS	Edinburgh Postnatal Depression Scale
TSH	Thyroid-stimulating hormone
FT3	Free triiodothyronine
FT4	Free tetraiodothyronine
APTT	Activated partial thromboplastin time
INR	International normalized ratio
CRP	C-reactive protein
DSM-5	*Diagnostic and Statistical Manual of Mental Disorders, Fifth Edition*
HIV	Human immunodeficiency virus
AIDS	Acquired immunodeficiency syndrome
EDTA	Ethylenediaminetetraacetic acid
CBC	Complete blood count
ACOG	American College of Obstetricians and Gynecologists
AAFP	American Academy of Family Physicians
AAP	American Academy of Pediatrics
PHQ-9	Patient Health Questionnaire-9
GAD-7	Generalized Anxiety Disorder Scale
GDPR	General Data Protection Regulation
WBC	White blood cells
RBC	Red blood cells
Hb	Hemoglobin
HCT	Hematocrit
PLT	Platelets
GABA	Gamma-aminobutyric acid
FDA	Food and Drug Administration
Hs-CRP	High-sensitivity C-reactive protein
IL-6	Interleukin-6
IL-1β	Interleukin-1β
BDNF	Brain-derived neurotrophic factor

## References

[1] Fonseca A., Ganho-Ávila A., Lambregtse-van den Berg M., Lupattelli A., Rodriguez-Muñoz M.d.l.F., Ferreira P., Radoš S.N., Bina R. (2020). Emerging Issues and Questions on Peripartum Depression Prevention, Diagnosis and Treatment: A Consensus Report from the Cost Action Riseup-PPD. J. Affect. Disord..

[2] American Psychiatric Association (2013). AP Diagnostic and Statistical Manual of Mental Disorders: DSM-5.

[3] ACOG Publications (2023). Treatment and Management of Mental Health Conditions During Pregnancy and Postpartum: ACOG Clinical Practice Guideline No. 5. Obs. Gynecol..

[4] Sharma R., Bansal P., Saini L., Sharma N., Dhingra R. (2024). Zuranolone, a Neuroactive Drug, Used in the Treatment of Postpartum Depression by Modulation of GABAA Receptors. Pharmacol. Biochem. Behav..

[5] The Global Burden of Disease: 2004 Update. https://www.who.int/publications/i/item/9789241563710.

[6] Levin G., Ein-Dor T. (2023). A Unified Model of the Biology of Peripartum Depression. Transl. Psychiatry.

[7] Zauderer C. (2009). Postpartum Depression: How Childbirth Educators Can Help Break the Silence. J. Perinat. Educ..

[8] Hutcherson T.C., Cieri-Hutcherson N.E., Gosciak M.F. (2020). Brexanolone for Postpartum Depression. Am. J. Health Syst. Pharm..

[9] Redei E.E., Ciolino J.D., Wert S.L., Yang A., Kim S., Clark C., Zumpf K.B., Wisner K.L. (2021). Pilot Validation of Blood-Based Biomarkers during Pregnancy and Postpartum in Women with Prior or Current Depression. Transl. Psychiatry.

[10] Kendler K.S., Gatz M., Gardner C.O., Pedersen N.L. (2006). A Swedish National Twin Study of Lifetime Major Depression. Am. J. Psychiatry.

[11] Burcusa S.L., Iacono W.G. (2007). Risk for Recurrence in Depression. Clin. Psychol. Rev..

[12] Luijendijk H.J., van den Berg J.F., Dekker M.J.H.J., van Tuijl H.R., Otte W., Smit F., Hofman A., Stricker B.H.C., Tiemeier H. (2008). Incidence and Recurrence of Late-Life Depression. Arch. Gen. Psychiatry.

[13] Altemus M., Sarvaiya N., Neill Epperson C. (2014). Sex Differences in Anxiety and Depression Clinical Perspectives. Front. Neuroendocr..

[14] Josefsson A., Sydsjö G. (2007). A Follow-up Study of Postpartum Depressed Women: Recurrent Maternal Depressive Symptoms and Child Behavior after Four Years. Arch. Womens Ment. Health.

[15] Milgrom J., Gemmill A.W., Bilszta J.L., Hayes B., Barnett B., Brooks J., Ericksen J., Ellwood D., Buist A. (2008). Antenatal Risk Factors for Postnatal Depression: A Large Prospective Study. J. Affect. Disord..

[16] O’Hara M.W. (2009). Postpartum Depression: What We Know. J. Clin. Psychol..

[17] Josefsson A., Angelsiöö L., Berg G., Ekström C.-M., Gunnervik C., Nordin C., Sydsjö G. (2002). Obstetric, Somatic, and Demographic Risk Factors for Postpartum Depressive Symptoms. Obs. Gynecol..

[18] Lindahl V., Pearson J.L., Colpe L. (2005). Prevalence of Suicidality during Pregnancy and the Postpartum. Arch. Womens Ment. Health.

[19] Slomian J., Honvo G., Emonts P., Reginster J.-Y., Bruyère O. (2019). Consequences of Maternal Postpartum Depression: A Systematic Review of Maternal and Infant Outcomes. Womens Health.

[20] Cepeda M.S., Kern D.M., Nicholson S. (2019). Treatment Resistant Depression in Women with Peripartum Depression. BMC Pregnancy Childbirth.

[21] Curry S.J., Krist A.H., Owens D.K., Barry M.J., Caughey A.B., Davidson K.W., Doubeni C.A., Epling J.W., Grossman D.C., US Preventive Services Task Force (2019). Interventions to Prevent Perinatal Depression: US Preventive Services Task Force Recommendation Statement. JAMA.

[22] Overview | Antenatal and Postnatal Mental Health: Clinical Management and Service Guidance | Guidance | NICE. https://www.nice.org.uk/guidance/cg192.

[23] Liu X., Wang S., Wang G. (2022). Prevalence and Risk Factors of Postpartum Depression in Women: A Systematic Review and Meta-Analysis. J. Clin. Nurs..

[24] ACOG Publications (2023). Screening and Diagnosis of Mental Health Conditions During Pregnancy and Postpartum: ACOG Clinical Practice Guideline No. 4. Obs. Gynecol..

[25] Cox J.L., Holden J.M., Sagovsky R. (1987). Detection of Postnatal Depression. Development of the 10-Item Edinburgh Postnatal Depression Scale. Br. J. Psychiatry.

[26] Tosto V., Ceccobelli M., Lucarini E., Tortorella A., Gerli S., Parazzini F., Favilli A. (2023). Maternity Blues: A Narrative Review. J. Pers. Med..

[27] Wallis A., Fernandez R., Florin O., Cherecheş R., Zlati A., Dungy C. (2012). Validation of a Romanian Scale to Detect Antenatal Depression. Cent. Eur. J. Med..

[28] Bergant A.M., Nguyen T., Heim K., Ulmer H., Dapunt O. (1998). German language version and validation of the Edinburgh postnatal depression scale. Dtsch. Med. Wochenschr..

[29] Vivilaki V.G., Dafermos V., Kogevinas M., Bitsios P., Lionis C. (2009). The Edinburgh Postnatal Depression Scale: Translation and Validation for a Greek Sample. BMC Public Health.

[30] O’Connor E., Rossom R.C., Henninger M., Groom H.C., Burda B.U. (2016). Primary Care Screening for and Treatment of Depression in Pregnant and Postpartum Women: Evidence Report and Systematic Review for the US Preventive Services Task Force. JAMA.

[31] Hewitt C.E., Gilbody S.M., Mann R., Brealey S. (2010). Instruments to Identify Post-Natal Depression: Which Methods Have Been the Most Extensively Validated, in What Setting and in Which Language?. Int. J. Psychiatry Clin. Pract..

[32] Online ACOG Publications (2018). ACOG Committee Opinion No. 757: Screening for Perinatal Depression. Obs. Gynecol..

[33] Orsolini L., Yılmaz-Karaman I.G., Bottaro M., Bellagamba S., Francesconi G., Volpe U. (2025). Preconception Paternal Mental Health History as Predictor of Antenatal Depression in Pregnant Women. Ann. Gen. Psychiatry.

[34] Levis B., Negeri Z., Sun Y., Benedetti A., Thombs B.D., DEPRESsion Screening Data (DEPRESSD) EPDS Group (2020). Accuracy of the Edinburgh Postnatal Depression Scale (EPDS) for Screening to Detect Major Depression among Pregnant and Postpartum Women: Systematic Review and Meta-Analysis of Individual Participant Data. BMJ.

[35] Shrestha S.D., Pradhan R., Tran T.D., Gualano R.C., Fisher J.R.W. (2016). Reliability and Validity of the Edinburgh Postnatal Depression Scale (EPDS) for Detecting Perinatal Common Mental Disorders (PCMDs) among Women in Low-and Lower-Middle-Income Countries: A Systematic Review. BMC Pregnancy Childbirth.

[36] JASP Team (2024). JASP (Version 0.19.3) [Computer Software].

[37] Dye C., Lenz K.M., Leuner B. (2022). Immune System Alterations and Postpartum Mental Illness: Evidence From Basic and Clinical Research. Front. Glob. Womens Health.

[38] Schiller C.E., Meltzer-Brody S., Rubinow D.R. (2015). The Role of Reproductive Hormones in Postpartum Depression. CNS Spectr..

[39] Skalkidou A., Hellgren C., Comasco E., Sylvén S., Sundström Poromaa I. (2012). Biological Aspects of Postpartum Depression. Womens Health.

[40] Galea L.A., Wide J.K., Barr A.M. (2001). Estradiol Alleviates Depressive-like Symptoms in a Novel Animal Model of Post-Partum Depression. Behav. Brain Res..

[41] Stoffel E.C., Craft R.M. (2004). Ovarian Hormone Withdrawal-Induced “Depression” in Female Rats. Physiol. Behav..

[42] Soares C.N., Frey B.N. (2010). Challenges and Opportunities to Manage Depression during the Menopausal Transition and beyond. Psychiatr. Clin..

[43] Ahokas A., Kaukoranta J., Wahlbeck K., Aito M. (2001). Estrogen Deficiency in Severe Postpartum Depression: Successful Treatment with Sublingual Physiologic 17beta-Estradiol: A Preliminary Study. J. Clin. Psychiatry.

[44] Gregoire A.J., Kumar R., Everitt B., Henderson A.F., Studd J.W. (1996). Transdermal Oestrogen for Treatment of Severe Postnatal Depression. Lancet.

[45] Amin Z., Canli T., Epperson C.N. (2005). Effect of Estrogen-Serotonin Interactions on Mood and Cognition. Behav. Cogn. Neurosci. Rev..

[46] Meltzer-Brody S., Colquhoun H., Riesenberg R., Epperson C.N., Deligiannidis K.M., Rubinow D.R., Li H., Sankoh A.J., Clemson C., Schacterle A. (2018). Brexanolone Injection in Post-Partum Depression: Two Multicentre, Double-Blind, Randomised, Placebo-Controlled, Phase 3 Trials. Lancet.

[47] Kroska E.B., Stowe Z.N. (2020). Postpartum Depression: Identification and Treatment in the Clinic Setting. Obs. Gynecol. Clin. N. Am..

[48] Sylvén S.M., Elenis E., Michelakos T., Larsson A., Olovsson M., Poromaa I.S., Skalkidou A. (2013). Thyroid Function Tests at Delivery and Risk for Postpartum Depressive Symptoms. Psychoneuroendocrinology.

[49] Schmidt P.M.d.S., Longoni A., Pinheiro R.T., Assis A.M.d. (2022). Postpartum Depression in Maternal Thyroidal Changes. Thyroid Res..

[50] Zhu J., Jin J., Tang J. (2022). Inflammatory Pathophysiological Mechanisms Implicated in Postpartum Depression. Front. Pharmacol..

[51] Lambert M., Gressier F. (2019). Inflammatory Biomarkers and Postpartum Depression: A Systematic Review of Literature. Can. J. Psychiatry.

[52] Worthen R.J., Beurel E. (2022). Inflammatory and Neurodegenerative Pathophysiology Implicated in Postpartum Depression. Neurobiol. Dis..

[53] Liu H., Zhang Y., Gao Y., Zhang Z. (2016). Elevated Levels of Hs-CRP and IL-6 after Delivery Are Associated with Depression during the 6 Months Post Partum. Psychiatry Res..

[54] Abedi P., Bovayri M., Fakhri A., Jahanfar S. (2018). The Relationship Between Vitamin D and Postpartum Depression in Reproductive-Aged Iranian Women. J. Med. Life.

[55] Robinson M., Whitehouse A.J.O., Newnham J.P., Gorman S., Jacoby P., Holt B.J., Serralha M., Tearne J.E., Holt P.G., Hart P.H. (2014). Low Maternal Serum Vitamin D during Pregnancy and the Risk for Postpartum Depression Symptoms. Arch. Womens Ment. Health.

[56] Albacar G., Sans T., Martín-Santos R., García-Esteve L., Guillamat R., Sanjuan J., Cañellas F., Gratacòs M., Cavalle P., Arija V. (2011). An Association between Plasma Ferritin Concentrations Measured 48 h after Delivery and Postpartum Depression. J. Affect. Disord..

[57] Wassef A., Nguyen Q.D., St-André M. (2019). Anaemia and Depletion of Iron Stores as Risk Factors for Postpartum Depression: A Literature Review. J. Psychosom. Obs. Gynaecol..

